# Functional Conservation of *Gsdma* Cluster Genes Specifically Duplicated in the Mouse Genome

**DOI:** 10.1534/g3.113.007393

**Published:** 2013-10-01

**Authors:** Shigekazu Tanaka, Youichi Mizushina, Yoriko Kato, Masaru Tamura, Toshihiko Shiroishi

**Affiliations:** Mammalian Genetics Laboratory, Genetic Strains Research Center, National Institute of Genetics, 1111 Yata, Mishima, Shizuoka 411-8450, Japan

**Keywords:** Gasdermin A, duplication, knockout, transgenic, alopecia

## Abstract

Mouse Gasdermin A3 (*Gsdma3*) is the causative gene for dominant skin mutations exhibiting alopecia. Mouse has two other *Gsdma3*-related genes, *Gsdma* and *Gsdma2*, whereas human and rat have only one related gene. To date, no skin mutation has been reported for human *GSDMA* and rat *Gsdma* as well as mouse *Gsdma* and *Gsdma2*. Therefore, it is possible that only *Gsdma3* has gain-of-function type mutations to cause dominant skin phenotype. To elucidate functional divergence among the *Gsdm*a-related genes in mice, and to infer the function of the human and rat orthologs, we examined *in vivo* function of mouse *Gsdma* by generating *Gsdma* knockout mice and transgenic mice that overexpress wild-type *Gsdma* or *Gsdma* harboring a point mutation (Alanine339Threonine). The *Gsdma* knockout mice shows no visible phenotype, indicating that *Gsdma* is not essential for differentiation of epidermal cells and maintenance of the hair cycle, and that *Gsdma* is expressed specifically both in the inner root sheath of hair follicles and in suprabasal cell layers, whereas *Gsdma3* is expressed only in suprabasal layers. By contrast, both types of the transgenic mice exhibited epidermal hyperplasia resembling the *Gsdma3* mutations, although the phenotype depended on the genetic background. These results indicate that the mouse *Gsdma* and *Gsdma3* genes share common function to regulate epithelial maintenance and/or homeostasis, and suggest that the function of human *GSDMA* and rat *Gsdma*, which are orthologs of mouse *Gsdma*, is conserved as well.

Gene duplication is a primary source of genetic diversity in evolution. Functional divergence of duplicated genes arises from differentiation in amino acid sequence and/or gene expression pattern between duplicated genes (Ohno 1970). Such differentiation is driven by the accumulation of mutations in the coding sequence and/or *cis*-regulatory elements during evolution (Lynch and Conery 2000; Zhang 2003). Elucidation of the diverged functions of the duplicated genes is important to understand how organisms acquire phenotypic diversity during evolution.

The Gasdermin (*Gsdm/GSDM*) gene family is composed of four paralogous genes, Gasdermin A (*Gsdma/GSDMA*), Gasdermin B (*GSDMB*), Gasdermin C (*Gsdmc/GSDMC*), and Gasdermin D (*Gsdmd/GSDMD*), in the mouse, rat, and human genomes. These genes were likely generated by two-round whole-genome duplications during vertebrate evolution (Tamura *et al.* 2007). The number of genes in each *Gsdm/GSDM* family differs among species. In mice, further tandem duplication occurred in the *Gsdma*, resulting in the formation of gene cluster: three *Gsdma*-related genes (*Gsdma*, *Gsdma2*, and *Gsdma3*) (Tamura *et al.* 2007) (Figure 1A). Phylogenetic analysis of the *Gsdma* cluster showed that human *GSDMA* has 87%, 74%, and 73% amino acid sequence similarity with mouse *Gsdma*, *Gsdma2*, and *Gsdma3*, respectively, indicating that human *GSDMA* is the counterpart of mouse *Gsdma* (Figure 1B) (Runkel *et al.* 2004). The *Gsdm/GSDM* family genes are differentially expressed in the epithelium from skin to gastrointestinal tract in a highly tissue-specific manner (Tamura *et al.* 2007). Although human *GSDMA* is mainly expressed in skin and stomach (Saeki *et al.* 2000), expression domains of mouse *Gsdma* cluster genes are divided into three compartments. *Gsdma* is expressed in the squamous epithelium from skin to the cardia of stomach, and *Gsdma2* and *Gsdma3* are specifically expressed in the epithelium of glandular stomach and skin, respectively (Runkel *et al.* 2004; Lunny *et al.* 2005; Tamura *et al.* 2007; Tanaka *et al.* 2007a) (Figure 1A).

**Figure 1:**
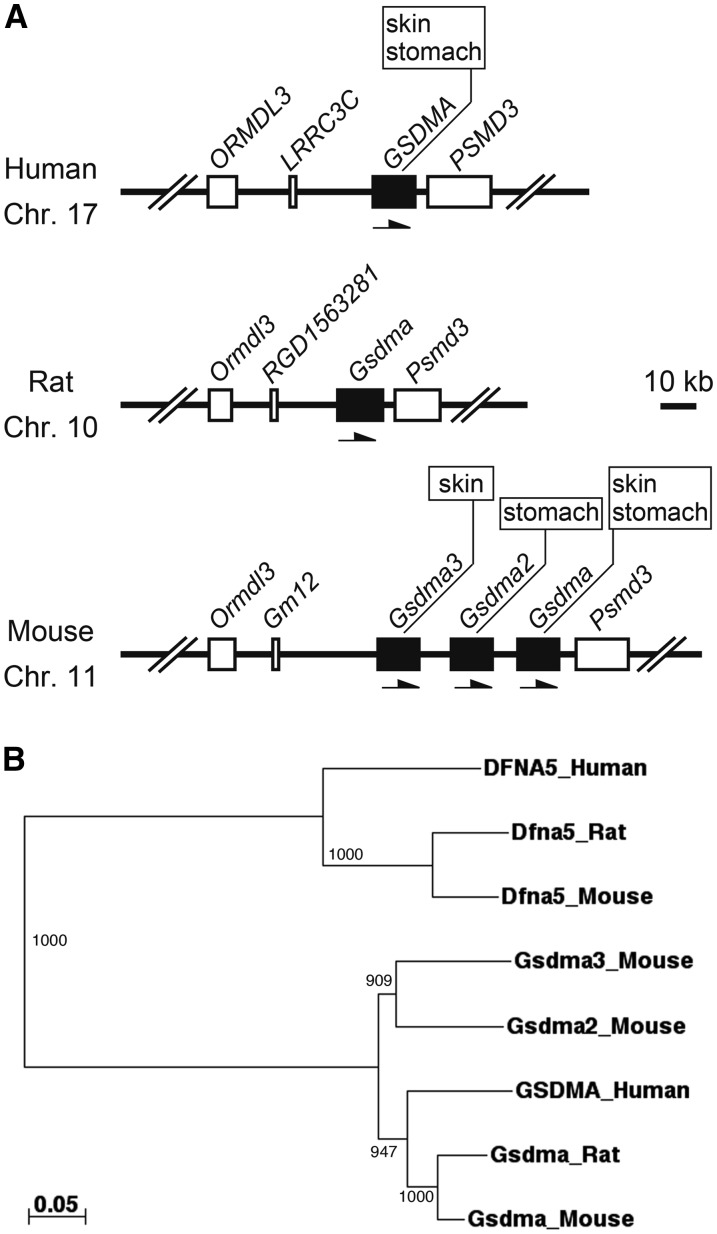
*Gsdma* orthologous genes in human, rat and mouse. (A) The genome structure of the *Gsdma* orthologous gene in human, rat and mouse was constructed based on Genome Reference Consortium Human Build 37, RGSC_v3.4, and Genome Reference Consortium Mouse Build 38, respectively. Transcriptional orientation of each gene is represented by an arrow. Expression domains of each *Gsdma* orthologous gene are shown in boxes. (B) Unrooted phylogenetic tree of *Gsdma* orthologous genes in human, rat, and mouse. The tree is constructed using a neighbor-joining method based on the multiple alignment generated by the ClustalW program. The numbers indicate the bootstrap values based on 1000 runs. DFNA5 orthologs are used as outgroup sequences. The scale bar indicates the number of amino acid substitutions per site.

The *Gsdm/GSDM* family genes encode 400−500 amino acid residues. A leucine-rich motif is well conserved in the C-terminus region across this family of genes, but there is no other known motif or domain (Tamura *et al.* 2007). The biologic function of the *Gsdm/GSDM* family genes initially was inferred from phenotypes of spontaneous and chemically-induced mouse mutants. To date, *Gsdma3* has been identified as the causative gene of eight mouse alopecia mutations, all of which exhibit dominant phenotype of hyperkeratosis and hair loss (Runkel *et al.* 2004; Lunny *et al.* 2005; Tanaka *et al.* 2007a; Sun *et al.* 2009; Li *et al.* 2010; Lei *et al.* 2011; Zhou *et al.* 2012). On the other hand, neither a mouse nor human mutation has been reported for other members of the *Gsdm/GSDM* family genes.

Altered expression patterns of the *GSDM* family genes in human cancer cell lines hinted at their cellular function *in vitro*. For instance, overexpression of human *GSDM* family genes, except for *GSDMB*, induces cell-growth inhibition in cancer cell lines (Saeki *et al.* 2007; Saeki *et al.* 2009). Gene expression of *GSDMA* and *GSDMD* was frequently suppressed, and *GSDMB* was overexpressed in cancer cell lines and/or cancer tissue specimens (Saeki *et al.* 2009; Komiyama *et al.* 2010). These studies, together with expression patterns of the *Gsdm/GSDM* family genes, suggest that these genes are involved in regulation of the epithelial cell proliferation and differentiation, but their functions *in vivo* are still poorly understood.

In this study, we intended to clarify the *in vivo* functions of the *Gsdm/GSDM* family genes, focusing on the mouse *Gsdma* cluster. We generated *Gsdma* knockout (KO) mice and transgenic (TG) mice with the wild-type or mutant-type *Gsdma* transgene. These results revealed that mouse *Gsdma* has a function similar to that of *Gsdma3*, suggesting that this function also is conserved in the human and rat orthologs.

## Materials and Methods

### Mice

The C57BL/6J (B6) strain originally was purchased from the Jackson Laboratory (Bar Harbor, ME) and is maintained at Genetic Strains Research Center, National Institute of Genetics (NIG, Mishima, Japan). ICR strain and (C57BL/6N × DBA/2N)F_1_ mice were purchased from CLEA Japan (Tokyo, Japan). Mice were housed in an SPF facility (12-hr light and dark cycles). The animal experiments in this study were approved by the Animal Care and Use Committee of NIG.

### Generation of *Gsdma* KO mice

We used the ploxFNFDT vector containing the floxed PGK-Neo-poly-A cassette, PGK-DTA-poly-A cassette, and two *loxP* sites that would enable global deletion of the *Gsdma* cluster genes in a future study. To construct the targeting vector, a 5.5-kb long arm containing exon 4–8, a 2.8-kb short arm composed of three DNA fragments containing exon 1–3 cloned from BAC RP23-395E DNA, and a LacZ-poly-A fragment were cloned from pCMV SPORT-βgal (Invitrogen Japan, Tokyo, Japan) by polymerase chain reaction (PCR). These DNA fragments were subcloned into the ploxFNFDT vector. To trace the expression of the *Gsdma* gene, the LacZ-poly-A fragment was inserted at the start codon in exon 2. This insertion disrupts the expression of the endogenous *Gsdma* gene but allows the expression of the *LacZ* gene controlled by the intact promoter activity of *Gsdma*. The targeting vector was linearized with *I-Sce I* and electroporated into TT2 embryonic stem (ES) cells, which are derived from a (B6 × CBA/J)F_1_ mouse (Yagi *et al.* 1993). G418-resistant ES cell clones were selected for homologous recombination by PCR and Southern blot analysis. Positive clones were aggregated with eight-cell embryos from ICR mice and transplanted into surrogate females. Male chimeras were mated with B6 females. Germline transmission of the targeting allele was confirmed by Southern blot analysis and PCR using the primer pairs: P1, 5′-AAATGGAGGGTGCAAACAAG-3′; and P2, 5′-GGGTCGTCAAGACCTGGTAA-3′. A digoxigenin-labeled probe for Southern blot analysis was synthesized by PCR using the primer pairs: F, 5′-GGATTGTGGTGGTAACGGTAG-3′; and R, 5′-CAGGACATCTTTGGGGAGTGC-3′. Signal was detected using alkaline phosphatase−conjugated antidigoxigenin antibody and CDP Star according to the manufacturer’s protocol (Roche Diagnostics Japan, Tokyo, Japan). Heterozygous mice were maintained by repeating backcrosses onto B6. We analyzed KO mice at N5 generations.

### Generation of keratin 5 (K5)-*Gsdma* TG mice

We obtained the pKM2L-phK5 vector containing the 6.3-kb human K5 promoter from RIKEN BioResource Center (BRC, Tsukuba, Japan). cDNAs were synthesized from total RNA derived from B6 skin using PrimeScript II (TAKARA, Otsu, Japan). We amplified full-length *Gsdma* from skin cDNA by PCR using KOD-plus DNA polymerase (TOYOBO CO., LTD, Osaka, Japan). PCR products were directly cloned into pCR-TOPOII vector (Invitrogen Japan). After confirming the wild-type *Gsdma* by sequencing, the open reading frame of *Gsdma* with the Kozak sequence was amplified by PCR using the following primer pairs with a restriction enzyme site: F, 5’-AAAAGATCTTAAGGCGCGCCACCATGTTTGAGAATGTCACCCG-3′; and R, 5′-AAAGTCGACTTAGGAATTCTTGCTTAGCA-3′. PCR products were digested at the *Bgl*II and *Sal*I sites. Digested PCR products were inserted into the pIRES2-EGFP vector. After confirming the open reading frame again by sequencing, the *Gsdma*-IRES2-EGFP fragment was replaced with the luciferase reporter gene in the pKM2L-phK5 vector at the *Afl*II and *Not*I sites. For the *Gsdma3^Rim3^*-type *Gsdma* mutation, site-directed mutagenesis was performed by PCR using KOD-plus taq polymerase. Inverted tail-to-tail primer pairs (F, 5′-CCGGACACGCTCCCCCACCTTT-3′; R, 5′-GGAGAGGCCAGCTTAGAGGGCAC-3′) containing a point mutation were phosphorylated with T4 kinase and ATP (Toyobo Co., Ltd, Osaka, Japan). PCR products were amplified using this primer pair and pCR-TOPOII vector containing *Gsdma* as a template and were directly self-ligated. The *Gsdma3^Rim3^*-type *Gsdma* fragment confirmed by sequencing was inserted into the pKM2L-phK5 vector in the same manner. The vector was linearized at the *I-Sce*I sites and microinjected into the pronuclei of fertilized eggs derived from (C57BL/6N × DBA/2N)F_1_ mice. TG mice were selected by PCR analysis using the primer pair for the human K5 promoter region (F, 5′-AGACTCAGCATAGGGCTGGA-3′; R, 5′-GGGAGAGGGTGGTATCCATT’) and the *Egfp* gene (F, 5′-ACGTAAACGGCCACAAGTTC-3′; R, 5′-AAGTCGTGCTGCTTCATGTG-3′). Several lines of TG mice selected by expression of the *Egfp* gene were maintained by repeating backcrosses onto B6.

### Histology

For detection of β-galactosidase (β-gal) activity, frozen skin sections (10 μm) were fixed with 0.2% glutaraldehyde for 2 min. After washing 3 times in phosphate-buffered saline, the sections were stained in X-gal solution at 37° for 6 hr. The sections were fixed with 4% paraformaldehyde and washed in phosphate-buffered saline. Nuclear fast red was used as the counterstain. Hematoxylin and eosin staining, and immunohistological analysis, were performed as previously reported (Tanaka *et al.* 2007b). The following primary antibodies were used in this study: Keratin 14 (K14, 1:50, Covance, Richmond, CA), K71 (1:3200, kindly provided by Y. Shimomura) (Aoki *et al.* 2001), Gsdma (1:50, Santa Cruz Biotechnology, Santa Cruz, CA), EGFP (1:50, Invitrogen Japan). These antibodies were detected by using appropriate secondary antibodies conjugated with Alexa Fluor 488 (Invitrogen Japan) or Alexa Fluor 594 (Invitrogen Japan). Nuclear staining was performed using To-pro3 (Invitrogen Japan).

## Results

### Generation of *Gsdma* KO mice

**Figure 2:**
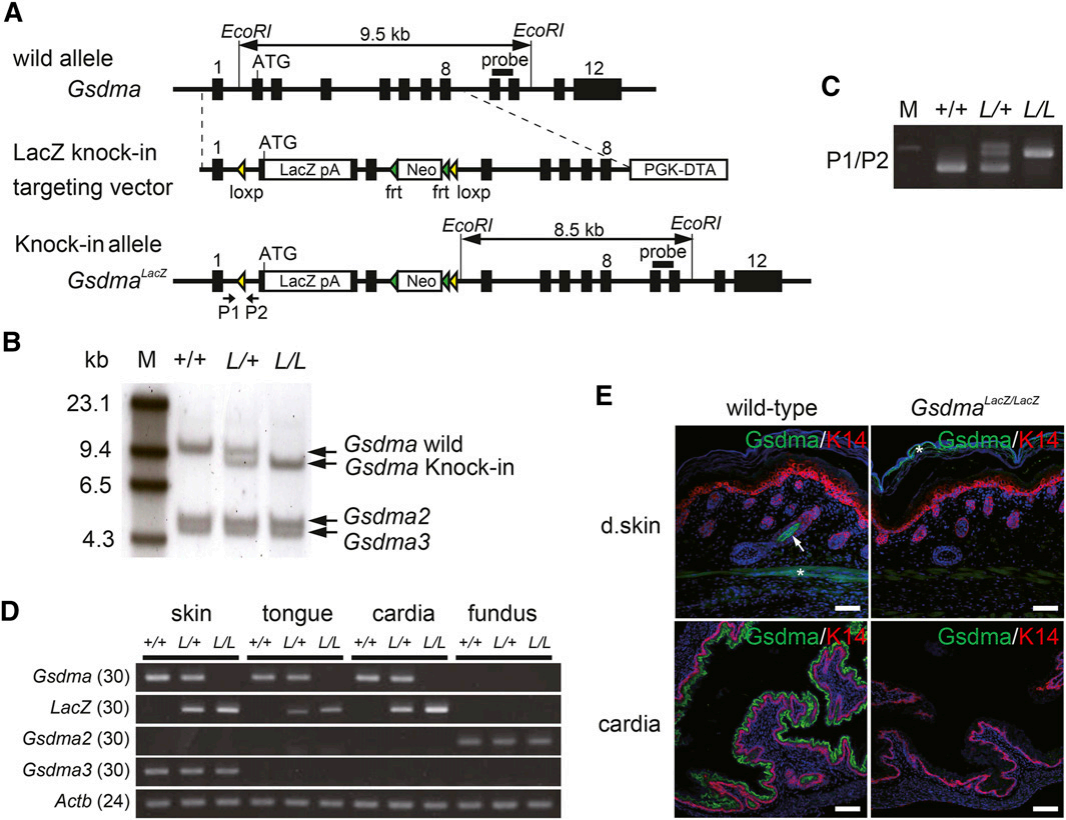
Generation and validation of the *Gsdma* KO mouse. (A) Schematic diagrams of wild-allele, *LacZ* knock-in targeting vector, and knock-in allele of the *Gsdma* gene. Exons are indicated as black boxes. *loxP* and *frt* sites are indicated as yellow and green triangles, respectively. The *LacZ*-pA fragment was inserted into the start codon in exon 2. *EcoRI* fragments and the probe for Southern blot analysis are indicated by horizontal bars with double arrows and horizontal bars. Location of primers used for genotyping are indicated by allows and labeled P1 and P2. (B) Southern blot analysis of *EcoRI* digested genomic DNA from wild-type (+/+), heterozygous (*L/+*), and homozygous (*L/L*) mice for the *LacZ* knock-in allele. The probe located in exon 9−10 detects 9.5- and 8.5-kb fragments of the wild and knock-in allele, respectively. The DNA fragments containing *Gsdma2* and *Gsdma3* are intact. M indicates a marker. (C) PCR analysis of genomic DNA from mice with each genotype. Genotype was determined by PCR using primers P1 and P2. This yielded fragments of 410 and 530 bp for the wild and knock-in allele, respectively. (D) Semiquantitative reverse-transcription PCR analysis of *Gsdma*, *Gsdma2*, *Gsdma3*, and *LacZ* genes using cDNAs prepared from skin, tongue, cardia, and gastric fundus of each genotype. Actin-beta (*Actb*) was amplified as a control. The number of PCR cycles is shown in parentheses. (E) Immunohistological detection of Gsdma protein in skin and stomach of wild-type and *Gsdma^LacZ/LacZ^* mice at E18.5. K14 was used as a marker for the basal cell layer. An arrow and asterisks indicate specific and nonspecific signal, respectively. Scale bar indicates 50 μm.

We generated *Gsdma* KO mice by inserting the *LacZ* reporter gene at the start codon of *Gsdma* (Figure 2A). We obtained three clones of targeted *Gsdma^LacZ/+^* ES cells by homologous recombination and then generated a *Gsdma* KO strain from one of these ES cell clones. Targeted alleles in the *Gsdma* KO mice were confirmed by Southern blot analysis and PCR genotyping (Figure 2, B and C). We confirmed the complete loss of the *Gsdma* transcript in homozygous *Gsdma^LacZ/LacZ^* mice by semiquantitative reverse-transcription PCR. In addition, *LacZ* mRNA expression was observed in heterozygous *Gsdma^LacZ/+^* and *Gsdma^LacZ/LacZ^* mice (Figure 2D). *Gsdma* was expressed in the skin, tongue, and cardia of wild-type (*Gsdma^+/+^*) mice, but it was not expressed in these tissues in *Gsdma^LacZ/LacZ^* mice (Figure 2D). Expression of the other *Gsdma* cluster genes, *Gsdma2* and *Gsdma3*, was not affected in the KO homozygotes (Figure 2D). Immunostaining using anti-Gsdma antibody revealed that Gsdma protein is localized in the inner root sheath (IRS) of hair follicles and suprabasal cell layers of the cardia epithelium in wild-type mice at embryonic day (E) 18.5, but it was not detected in *Gsdma^LacZ/LacZ^* mice (Figure 2E). These results demonstrated that *Gsdma* is completely and specifically targeted in the *Gsdma* KO allele.

### Spatial expression of *Gsdma* and *Gsdma3*

**Figure 3:**
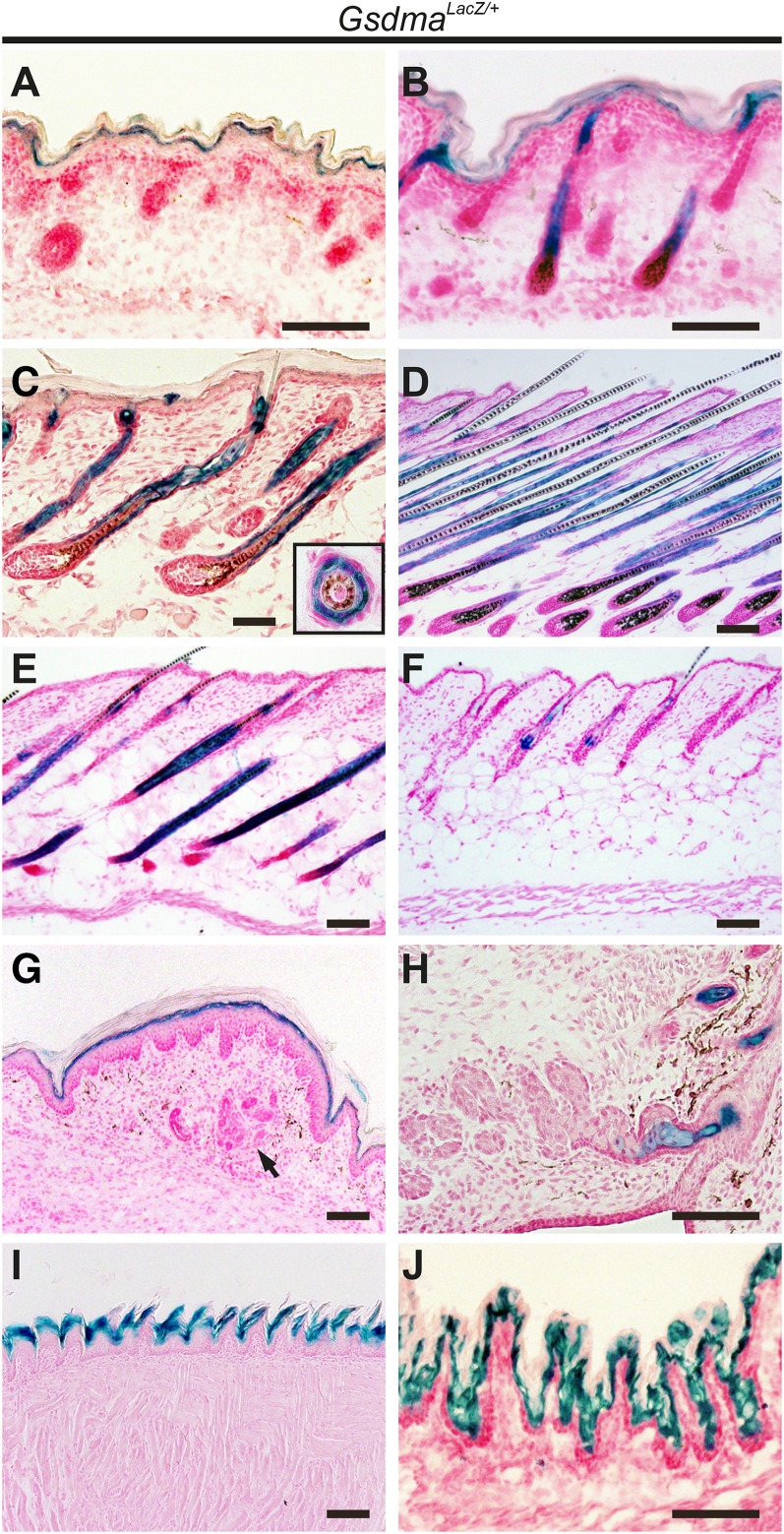
Expression of *LacZ* controlled by *Gsdma* promoter during epidermal development. X-gal−stained sections were made from *Gsdma^LacZ/+^* skin collected during the developmental stage at E18.5 (A), anagen at P1 (B), and P5 (C); catagen at P13 (D) and P19 (E); and telogen at P19 (F). The same sections were made for footpad at P11 (G), meibomian gland at P5 (H), tongue epithelium at P9 (I) and cardia of stomach at P1 (J) in *Gsdma^LacZ/+^* mice. An inset in (C) shows a cross section of a hair follicle at the same stage of development. An arrow (G) indicates sweat glands.

In neonatal mouse skin, both the *Gsdma* and *Gsdma3* genes are expressed (Runkel *et al.* 2004; Lunny *et al.* 2005; Tanaka *et al.* 2007a). However, the spatial distribution of the mRNA and protein of both *Gsdma* and *Gsdma3* remains elusive due to the high similarity in the nucleotide and amino acid sequences of the two genes (Runkel *et al.* 2004; Lunny *et al.* 2005; Tanaka *et al.* 2007a). To clarify the spatial distribution of endogenous *Gsdma* expression, we analyzed *LacZ* reporter expression in *Gsdma^LacZ/+^* mice. X-gal staining by β-gal activity was observed in differentiated epithelium derived from ectoderm such as skin, foot pad (except for sweat gland), meibomian gland, tongue, and the cardia region of the stomach (Figure 3, A−J). In the embryonic epidermis, strong expression was detected in the suprabasal cell layer (Figure 3A). In the neonatal epidermis, expression was especially strong in the IRS of hair follicles compared with the suprabasal cell layer (Figure 3, B and C). During the first hair cycle, β-gal activity was consistently detected in IRS. These results are consistent with our previous data obtained by *in situ* hybridization using probes for *Gsdma* and *Gsdma3* mRNA (Tanaka *et al.* 2007a).

**Figure 4:**
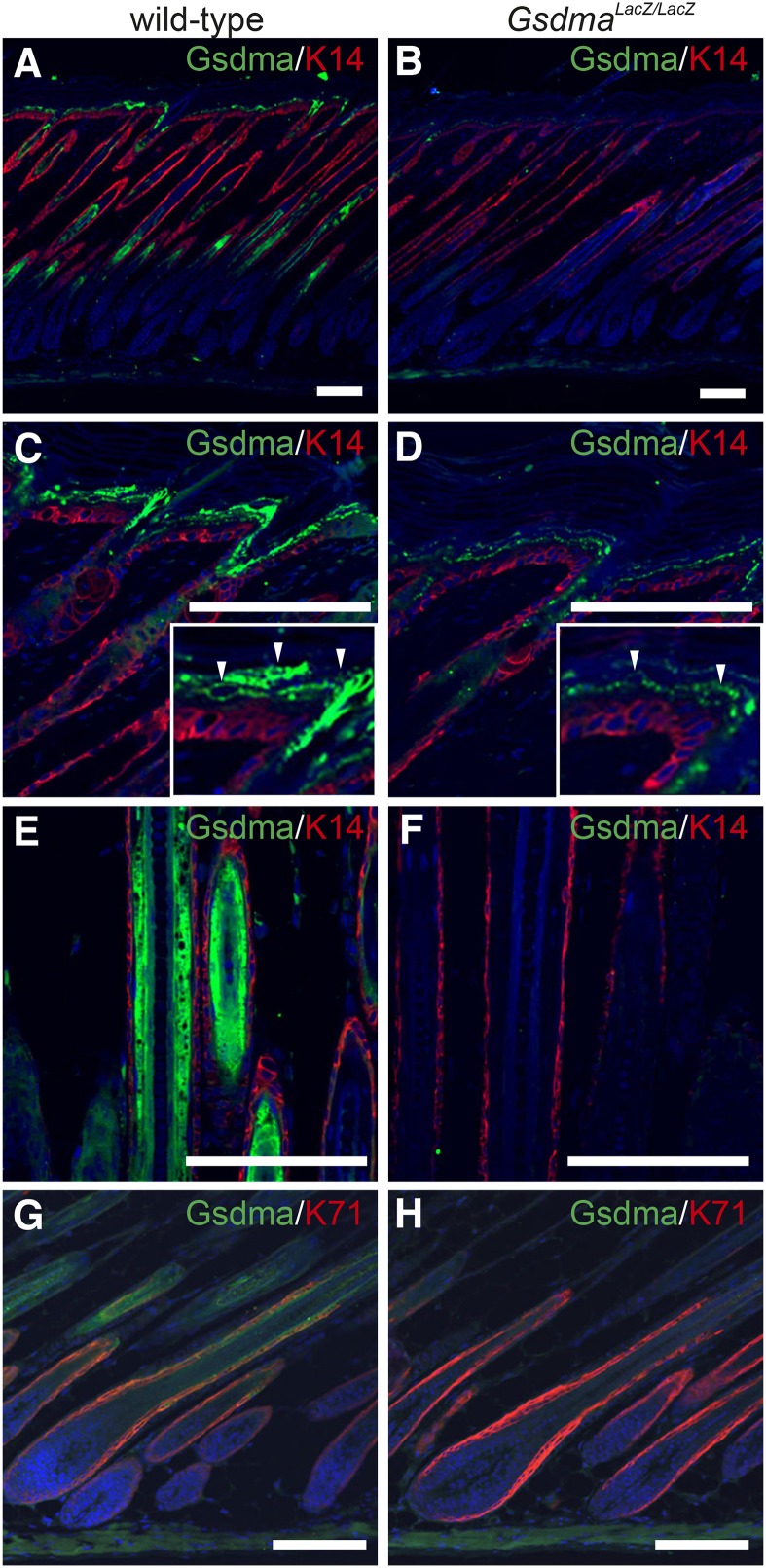
Distribution of Gsdma and Gsdma3 proteins. Immunohistological detection of Gsdma and Gsdma3 proteins in skin of wild-type (A, C, E, and G) and *Gsdma^LacZ/LacZ^* (B, D, F, and H) mice at P8. Magnified images are provided to show Gsdma protein (arrowheads) in the suprabasal cell layer. K14 and K71 were used as a marker for the basal cell layer and IRS, respectively. Scale bars are 25 μm.

Next, to analyze the spatial distribution of Gsdma3 protein in the epidermis, we carried out immunohistochemistry using anti-Gsdma antibody, which cross-reacts with Gsdma3 protein (Supporting Information, Figure S1). The signals were detected in both the suprabasal cells and IRS of wild-type epidermis (Figure 4, A, C, E, and G) at postnatal day (P) 8. Unexpectedly, in the KO epidermis, the signals were weakly located only in the suprabasal cell layer (Figure 4, B and D) but not in the IRS of hair follicles (Figure 4, B, F, and H). The expression level of *Gsdma3* in the suprabasal cell layer was the same in the wild-type and the KO epidermis at P5 by quantitative PCR analysis (Figure S2). All these data clearly indicated that *Gsdma* is expressed both in the suprabasal cells and IRS, whereas *Gsdma3* is predominantly expressed in the suprabasal cells.

### Normal development and homeostasis in *Gsdma* KO skin

**Figure 5:**
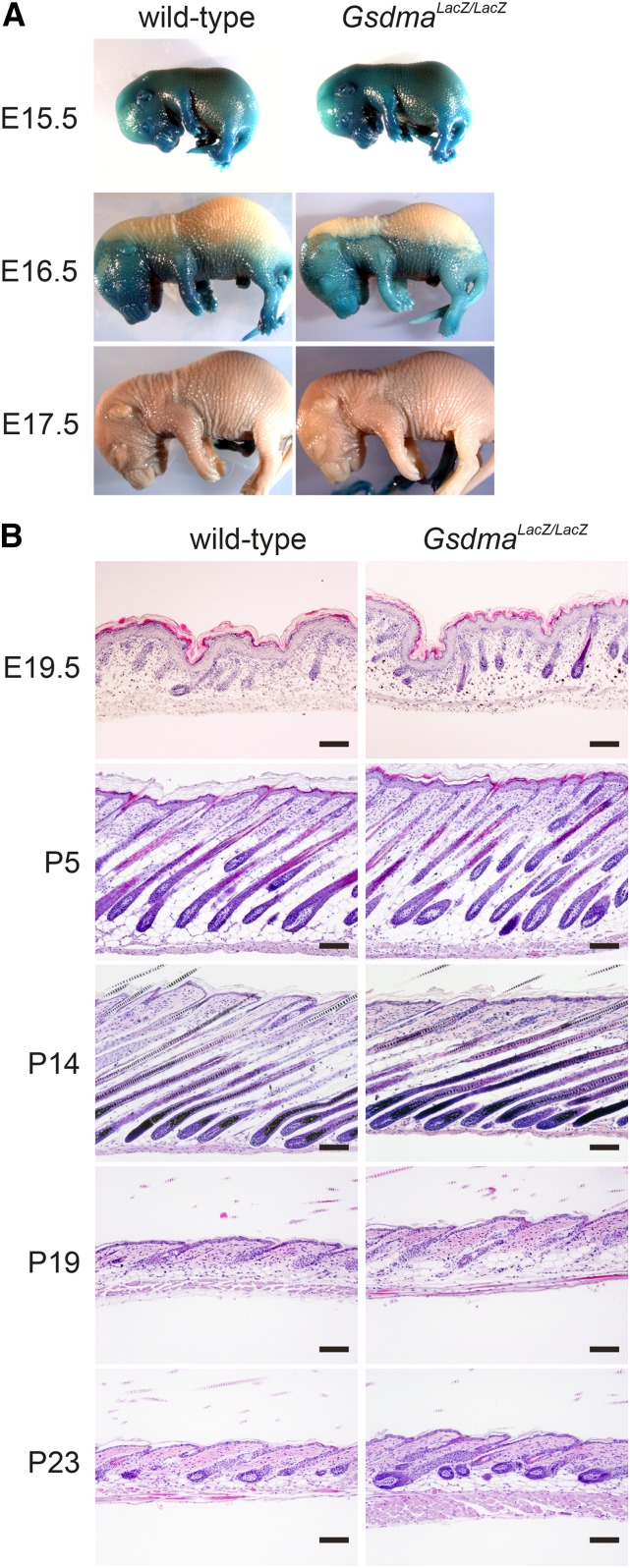
Phenotype of *Gsdma* KO mouse. (A) Result of skin permeability assay in wild-type and *Gsdma^LacZ/LacZ^* mice at the ages indicated. (B) HE stained sections of skin during the first hair cycle in wild-type and *Gsdma^LacZ/LacZ^* mice. Scale bars are 100 μm.

*Gsdma* KO homozygotes (*Gsdma^LacZ/LacZ^*) were born at the expected Mendelian ratio following intercrossing of *Gsdma^LacZ/+^* mice (data not shown). No obvious developmental abnormality was observed in the *Gsdma^LacZ/LacZ^* mice. Skin permeability assay showed no difference in the terminal differentiation of epidermal cells at the embryonic stage between wild-type and KO homozygotes, although *Gsdma* expression starts at the embryonic stage (Figure 5A). We next performed a comprehensive histologic analysis of skin during the first hair cycle. Again, no apparent morphologic difference was observed between wild-type and *Gsdma^LacZ/LacZ^* mice (Figure 5B). Finally, we extended the observation time period to 1 month after birth. Immunohistochemistry with anti-Keratin 14 antibody reacting to basal cells, anti-keratin 10 antibody reacting to suprabasal cells, and anti-Filaggrin antibody reacting to cornified cells, detected no difference in the signals of these antibodies for epidermal cells from wild-type and *Gsdma^LacZ/LacZ^* mice (Figure S3). Moreover, 1-yr-old *Gsdma^LacZ/LacZ^* mice did not exhibit any morphologic abnormalities such as alopecia or skin tumor development (data not shown). These results indicate that *Gsdma* is not essential for differentiation of epidermal cells and maintenance of the hair cycle.

### K5-*Gsdma*-A339T TG mice exhibit alopecia similar to the *Gsdma3* mutant

**Figure 6:**
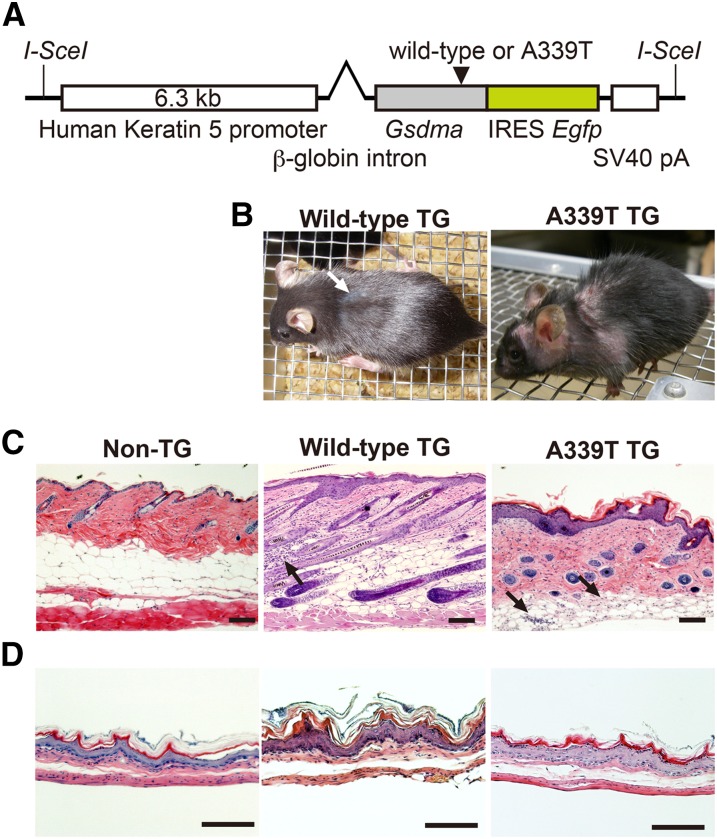
Phenotype of K5-*Gsdma* TG mouse. (A) Schematic diagrams of *Gsdma* TG vector construct with human K5 promoter. *Gsdma* in the construct is either the wild-type or has the *Rim3*-type mutation, A339T. (B) Macroscopic phenotypes of mice with wild-type or A339T mutant *Gsdma* transgenes at 3 months of age. A white arrow indicates patchy rough coat. Hematoxylin and eosin−stained sections of skin (C) and cardia (D) from control mouse (Non-TG), wild-type and A339T TG mice at 3 months of age. Inflammatory cells are present in the dermal fat layer (Arrows in C). Scale bars are 100 μm.

We generated TG mice that express a mutant form of *Gsdma*. This transgene has the *Gsdma3^Rim3^*-type point mutation, A339T, and its expression in skin is driven by human K5 promoter (Figure 6A). In parallel, we also generated another type of TG mouse that expresses wild-type *Gsdma* with the aim of examining the effect of *Gsdma* overexpression in skin.

We obtained eight founder mice that expressed the mutant (A339T) *Gsdma* transgene and two founder mice that expressed the wild-type *Gsdma* transgene. Irrespective of the type of transgene construct, all these founder mice exhibited no apparent abnormalities in their skin. To examine the genetic background effect on the phenotype, we backcrossed these founder mice onto B6 mice. After three generations of backcross, one line with the wild-type *Gsdma* transgene started to exhibit a partial rough coat phenotype, and one line with the mutant form (A339T) of the *Gsdma* transgene started to exhibit alopecia resembling the *Gsdma3* mutants (Figure 6B). Similar skin phenotypes to these two lines were observed in subsequent backcross generations. Notably, these phenotypes are observed only in less than 10% of the progeny with the hemizygous transgene. We further carried out histological analysis of abnormal epidermis from the descendant TG mice with the hemizygous transgenes. Epidermal hyperplasia was observed in both lines of mice (Figure 6C). These phenotypes were observed in restricted regions in which patchy EGFP expression was observed. This patchwork epidermal hyperplasia was more severe in the mouse line with the mutant (A339T) transgene than in the mouse line with the wild-type transgene (Figure 6C). Inflammatory cells were infiltrated in the dermis of both TG lines (Figure 6C). The complete loss of hair follicles was observed in 1-yr-old mice with the mutant transgene (Figure S4). These skin abnormalities resemble those of the *Gsdma3* mutant mice (Runkel *et al.* 2004; Lunny *et al.* 2005; Tanaka *et al.* 2007a; Sun *et al.* 2009; Li *et al.* 2010; Lei *et al.* 2011; Zhou *et al.* 2012). Epithelial hyperplasia also was observed in the stomach of both TG lines (Figure 6D) and may be due to the human K5 promoter in the transgene vector construct, which is active in the basal cell layer of stratified squamous epithelium from skin to forestomach.

**Figure 7:**
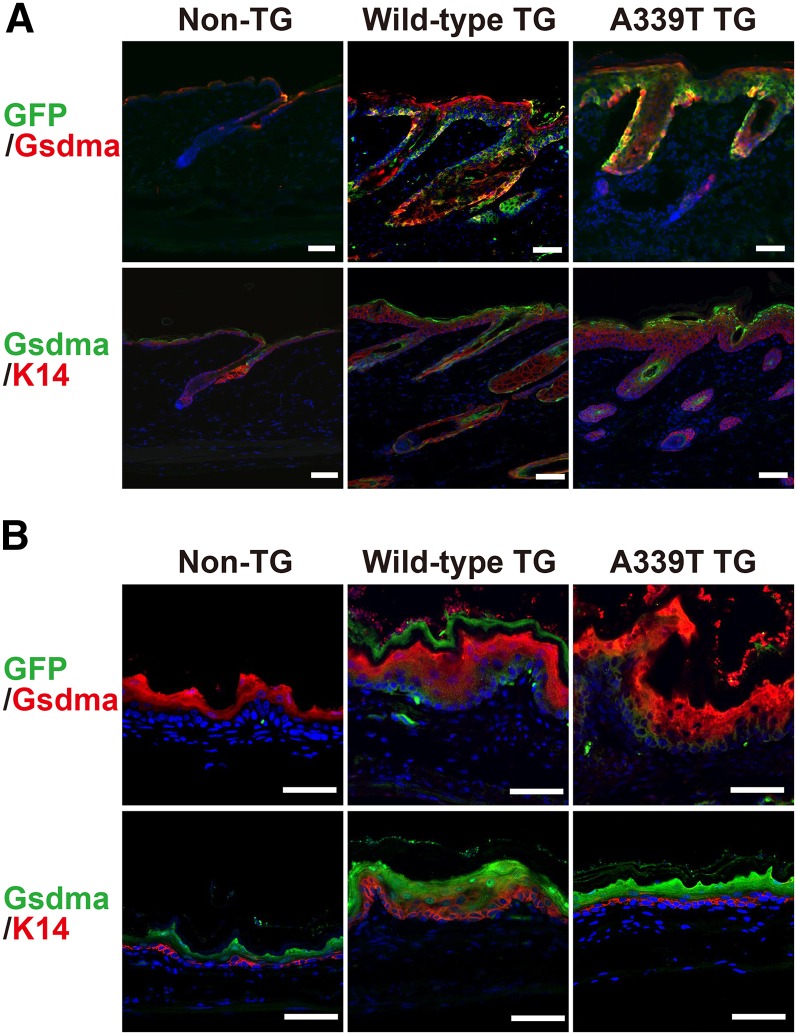
Mice with K5-*Gsdma* transgene exhibit epidermal hyperplasia. Immunohistological staining of skin (A) and cardia (B) from control mouse (Non-TG), and mice with wild-type and A339T *Gsdma* transgene, at 3 months of age. The sections were stained with anti-GFP and anti-Gsdma antibodies or anti-Gsdma and anti-K14 antibodies. Nuclei were stained with ToPro3. Scale bars are 50 μm.

Next, we examined expression of the transgene in the affected cells by immunohistochemistry with EGFP and anti-Gsdma antibody. Overlapping patchy signals of EGFP and Gsdma protein were observed in the basal cell layer of the skin and cardia of both TG lines (Figure 7, A and B). Immunohistochemistry with K14 antibody revealed that the epidermal hyperplasia is specific to the basal cell layer in the skin and cardia of both TG lines (Figure 7, A and B). These results clearly demonstrated that the mutant form (A339T) of the *Gsdma* transgene and overexpression of the wild-type *Gsdma* transgene directly caused the epithelial hyperplasia.

## Discussion

The *Gsdma* KO mice are viable and show no visible phenotype, indicating that *Gsdma* is not an essential gene and is not involved in differentiation of epidermal cells and maintenance of the hair cycle. By contrast, the phenotype of the TG mice indicates that *Gsdma* has the same function to regulate epithelial cell proliferation and/or epithelial maintenance as the mouse paralogous gene, *Gsdma3*. It is notable that the alopecia started to appear only in the progeny obtained by backcrossing of the original TG mice with the mixed genetic background onto the B6 strain. This may be the reason why the *Gsdma* mutations have not been detected in large-scale and chemically induced mutagenesis projects, since these mutagenesis projects have mostly used mice with heterozygous F_1_ backgrounds.

The present study, in which we used *Gsdma* KO mice, allowed us to observe the precise expression pattern of *Gsdma3* without influence of *Gsdma*. Our findings clearly verify that *Gsdma3* is predominantly expressed in the suprabasal cells of the interfollicular epidermis, but not in the follicular epithelium. Consistent with this result, *Gsdma3* mutant mice initially exhibit hyperplasia in the interfollicular epidermis, but not in the follicular epithelium (Tanaka *et al.* 2007a).

The loss-of-function type mouse mutants of *Gsdm* family genes, such as the *Gsdmd* KO mice in our previous study and the *Gsdma* KO mice in this study, show neither morphologic anomaly such as epithelial hyperplasia nor tumor development (Fujii *et al.* 2008). The failure to identify a visible phenotype in the KO mice may be due to functional redundancy with the other genes. Alternatively, one explanation for the absence of phenotype in the KO mice might be that the members of the *Gsdm* family control susceptibility to environmental factors, such as allergens and infectious agents, or physical stress. It is known that newly duplicated genes in the mouse genome significantly enrich the functional category involved in immune defense, olfaction and drug metabolism, which are of medical importance (Cheung *et al.* 2003). In this regard, implication of susceptibility to environmental factors has been reported for function of the *Gsdm* family genes. *Gsdma* expression is down-regulated in Grainyhead-like 3 (*Grhl3*) KO mice, and the binding motif of the Grhl3 protein is conserved upstream of the transcription initiation site of the *Gsdm*a*/GSDMA* gene in mouse and human, suggesting that *Gsdma/GSDMA* is the target gene of *Grhl3* (Yu *et al.* 2006). *Grhl3* is a master regulator of epidermal terminal differentiation, and it controls wound response in mice and drosophila (Mace *et al.* 2005; Ting *et al.* 2005; Yu *et al.* 2006). Furthermore, it was recently reported that polymorphisms with *GSDMA* and *GSDMB* loci are associated with asthma, atopy and intermediate phenotypes such as elevated immunoglobulin E (Lluis *et al.* 2011; Yu *et al.* 2011). Thus, we infer that members of the *Gsdm* family genes play some role in stress responses to environmental factors. It would be of interest to test whether the KO mice carrying the *Gsdma* and *Gsdmd* genes show some new phenotypes under stress conditions in future studies.

The molecular basis of epidermal hyperplasia observed in the skin of mice with the *Gsdma* transgenes is unclear, but it may be related to the inflammation observed in the skin of those mice. Transforming-growth factor β (Tgfβ) is a key cytokine involved in apoptosis and inflammation (Letterio and Roberts 1998; Siegel and Massague 2003). Overexpression of *Tgfβ1* in the skin also causes inflammatory skin abnormalities (Wang *et al.* 1999; Liu *et al.* 2001; Li *et al.* 2004; Lu *et al.* 2004; Fitch *et al.* 2009). Moreover, *TGFβ* up-regulates *GSDMA* expression through *lmo1*, and the resultant *GSDMA* overexpression induces apoptosis in human cancer cell lines (Saeki *et al.* 2007). This finding is consistent with the fact that increased apoptotic cells are observed in the hair follicles of not only *Tgfβ1* TG mice but also *Gsdma3* mutant mice (Liu *et al.* 2001; Lei *et al.* 2011). These findings suggest that *Gsdma/GSDMA* has a role in regulating inflammation downstream of *TGFβ/Tgfβ in vivo*.

The dominant *Gsdma3* alleles in mice are categorized as nonsense- and missense-mutations (Runkel *et al.* 2004; Lunny *et al.* 2005; Tanaka *et al.* 2007a; Sun *et al.* 2009; Li *et al.* 2010; Lei *et al.* 2011; Zhou *et al.* 2012). A mutant allele, *Gsdma3^Dfl^*, exhibits alopecia resembling *Gsdma3^Rim3^* (Lunny *et al.* 2005). This mutation has a B2 element insertion in exon 7, and generates mRNA with a stop codon, which results in a C-terminally truncated Gsdma protein (Lunny *et al.* 2005). Deafness autosomal-dominant 5 (*DFNA5*) belongs to the *DFNA5-Gsdm* family. The N-terminus region of DFNA5 has the same amino acid sequence as the Gsdm family proteins and is referred to as the DFNA5-Gasdermin domain (Delmaghani *et al.* 2006; Tamura *et al.* 2007). Germline mutations in *DFNA5* have been discovered in human families and cause autosomal-dominant hearing loss (Van Laer *et al.* 1998; Yu *et al.* 2003; Cheng *et al.* 2007). In many cases, the *DFNA5* mutations lead to exon 8 skipping, resulting in frameshift and premature truncation of the protein (Van Laer *et al.* 1998; Yu *et al.* 2003; Cheng *et al.* 2007). It was reported that *Dfna5* KO mice, which have a deletion of exon 8 mimicking the human mutation, produce no Dfna5 protein through nonsense-mediated RNA decay (Van Laer *et al.* 2005). *Dfna5* KO mice display no hearing loss and do not mimic human hearing loss caused by mutations in *DFNA5* (Van Laer *et al.* 2005). However, the expression of mutant truncated DFNA5 protein leads to cell-cycle arrest in fission yeast (Gregan *et al.* 2003), and the expression of an aberrant N-terminus truncated form of DFNA5 causes apoptosis in cultured human cell lines (Op de Beeck *et al.* 2011). Therefore, it is inferred that DFNA5-associated hearing loss is caused by a gain-of-function mutation, but not by haplo-insufficiency (Op de Beeck *et al.* 2011). Taken together, although the physiologic function of full-length proteins of the *Gsdma/GSDMA* and *Dfna5* genes remains elusive, the expression of the truncated N-terminus region of the DFNA5-Gsdm domain affects cell proliferation, leading to dominant phenotypes in skin and inner ear.

## Supplementary Material

Supporting Information
