# Supplementary material for: Functional Conservation of Gsdma Cluster Genes Specifically Duplicated in the Mouse Genome
Source: G3 (Bethesda). 2013 Oct 1;3(10):1843–50. doi: 10.1534/g3.113.007393 (PMC3789809; doi:10.1534/g3.113.007393)
Supplement: Supporting Information [file supp_3_10_1843__index.html]

Functional Conservation of Gsdma Cluster Genes Specifically Duplicated in the Mouse Genome — Supporting Information 

# Functional Conservation of *Gsdma* Cluster Genes Specifically Duplicated in the Mouse Genome

## Supporting Information for Tanaka *et al.*, 2013

**Files in this Data Supplement:**

- Supporting Information - Figures S1-S4 (PDF, 2 MB)
- Figure S1 - Characterization of polyclonal Gsdm antibody (PDF, 1 MB)
- Figure S2 - Relative expression levels of *Gsdma* and *Gsdma3* genes in *GsdmaLacZ/LacZ* skin (PDF, 505 KB)
- Figure S3 - The expression of epidermal differentiation markers (PDF, 3 MB)
- Figure S4 - Phenotypes of a mouse with K5-*Gsdma* (A339T) transgene at 1 year of age. (PDF, 3 MB)
