# Supplementary material for: Functional Conservation of Gsdma Cluster Genes Specifically Duplicated in the Mouse Genome
Source: G3 (Bethesda). 2013 Oct 1;3(10):1843–50. doi: 10.1534/g3.113.007393 (PMC3789809; doi:10.1534/g3.113.007393)
Supplement: Supporting Information [file supp_g3.113.007393_007393SI.pdf]

G3 · Genes | Genomes | Genetics

Functional conservation of *Gsdma* cluster genes specifically duplicated in the mouse genome

Shigekazu Tanaka[*,1], Youichi Mizushina[*], Yoriko Kato[*], Masaru Tamura[*] and Toshihiko Shiroishi[*]

[*]Mammalian Genetics Laboratory, Genetic Strains Research Center, National Institute of Genetics. 1111 Yata, Mishima, Shizuoka 411-8450, Japan
[1] Present address: Juntendo Casualty Center, 1129 Nagaoka, Izunokuni, Shizuoka 410-2211, Japan

DOI: 10.1534/g3.113.007393

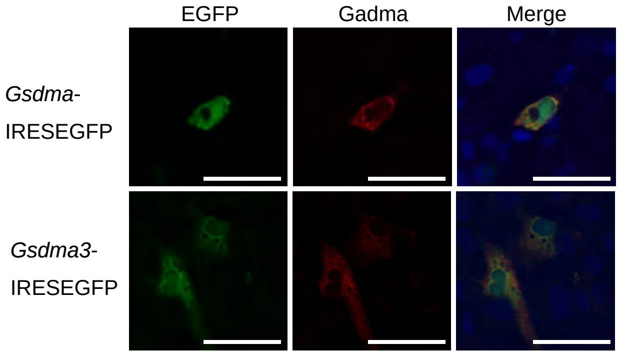

Figure S1 Characterization of polyclonal Gsdm antibody. Immunostaining of Gsdma-IRES-EGFP or Gsdma3-IRES-EGFP expressing Cos7 cells. The cells were fixed with 10% formaldehyde, permeabilized with 0.5% Triton-X 100, and immunostained with antibody against Gsdma. Nuclei were stained with ToPro3. This antibody cross-reacts with Gsdma3 protein. Scale bars are 50 µm.

Expression levels at P5 epidermis

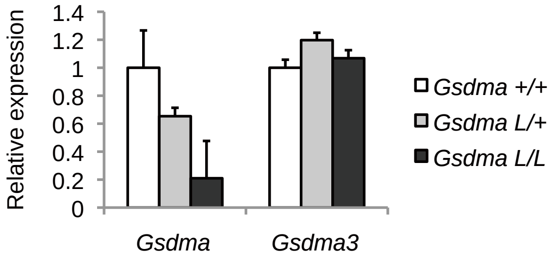

Figure S2 Relative expression levels of *Gsdma* and *Gsdma3* genes in *Gsdma*$^{LacZ/LacZ}$ skin. Real-time quantitative PCR was performed with TaqMan universal PCR Master Mix reagent using an ABI Prism 7700 (Applied Biosystems, Tokyo, Japan). cDNA was synthesized from 1 µg of DNase-treated total RNA from skin of wild-type (+/+), heterozygous (*L*/+) and homozygous (*L*/*L*) mice using SuperScriptTM III (Invitrogen Japan, Tokyo, Japan). Actb was used for normalization. Real-time quantitative PCR analysis confirmed the unchanged relative expression levels of the *Gsdma3* gene between wild-type and *Gsdma*$^{LacZ/LacZ}$ mice at P5.

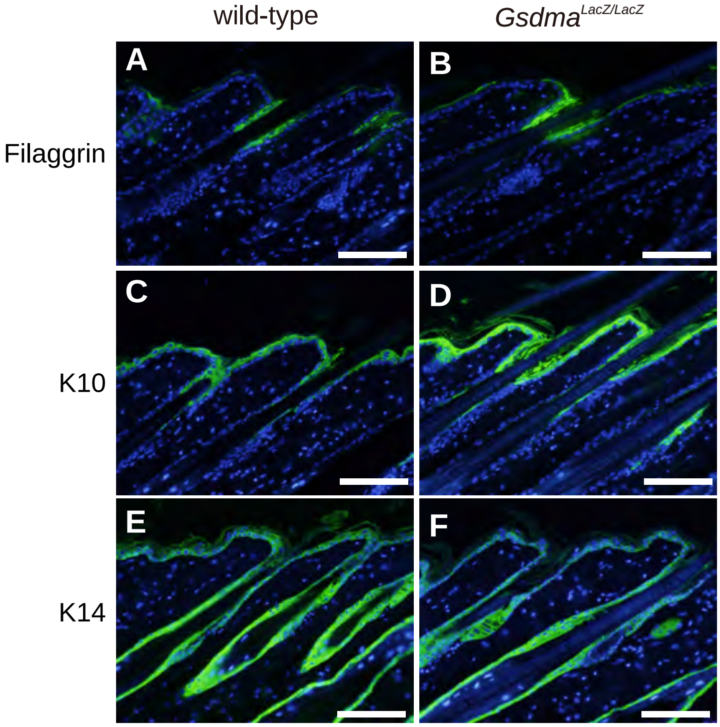

Figure S3 The expression of epidermal differentiation markers. Skin sections of wild-type (**A**, **C** and **E**) and *Gsdma*$^{LacZ/LacZ}$ mice (**B**, **D** and **F**) at 1 month of age. Filaggrin (1:100, Covance, Richmond, CA, USA) was used as a marker for the cornified cell layer (**A** and **B**). K10 (1:50, Covance) was used as a marker for the granular cell layer (**C** and **D**). K14 was used as a marker for the basal cell layer (**E** and **F**). Nuclear staining was performed by DAPI (Invitrogen Japan). Scale bars are 100 μm.

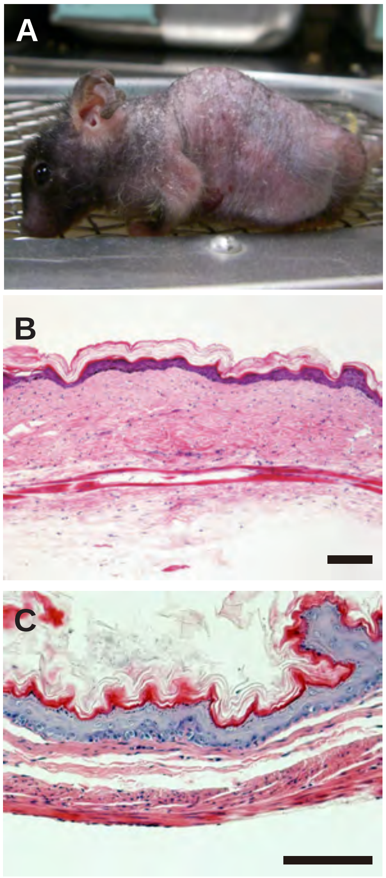

Figure S4. Phenotypes of a mouse with K5-*Gsdma* (A339T) transgene at 1 year of age. Macroscopic phenotype of a K5-*Gsdma* (A339T) transgenic mouse at 1 year of age (**A**). HE stained sections of skin (**B**) and cardia (**C**) of a K5-*Gsdma* (A339T) transgenic mouse at 1 year of age. Scale bars are 100 µm.
