# Supplementary material for: Functional Conservation of Gsdma Cluster Genes Specifically Duplicated in the Mouse Genome
Source: G3 (Bethesda). 2013 Oct 1;3(10):1843–50. doi: 10.1534/g3.113.007393 (PMC3789809; doi:10.1534/g3.113.007393)
Supplement: Supporting Information [file supp_g3.113.007393_FigureS1.pdf]

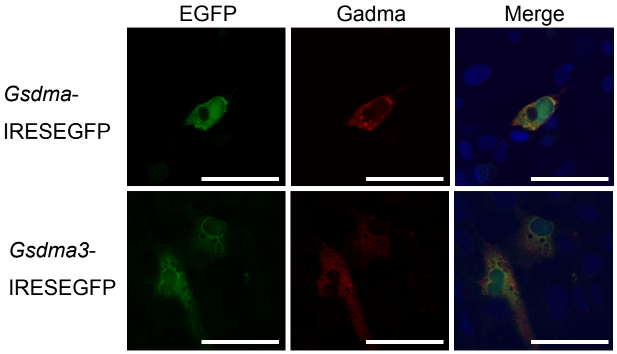

Figure S1 Characterization of polyclonal Gsdm antibody. Immunostaining of Gsdma-IRES-EGFP or Gsdma3-IRES-EGFP expressing Cos7 cells. The cells were fixed with 10% formaldehyde, permeabilized with 0.5% Triton-X 100, and immunostained with antibody against Gsdma. Nuclei were stained with ToPro3. This antibody cross-reacts with Gsdma3 protein. Scale bars are 50 µm.
