# Supplementary material for: Functional Conservation of Gsdma Cluster Genes Specifically Duplicated in the Mouse Genome
Source: G3 (Bethesda). 2013 Oct 1;3(10):1843–50. doi: 10.1534/g3.113.007393 (PMC3789809; doi:10.1534/g3.113.007393)
Supplement: Supporting Information [file supp_g3.113.007393_FigureS2.pdf]

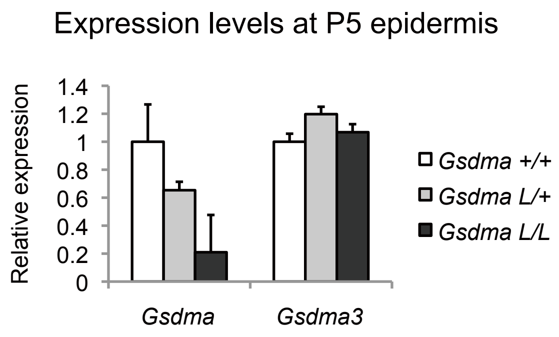

Figure S2 Relative expression levels of *Gsdma* and *Gsdma3* genes in *Gsdma*$^{LacZ/LacZ}$ skin. Real-time quantitative PCR was performed with TaqMan universal PCR Master Mix reagent using an ABI Prism 7700 (Applied Biosystems, Tokyo, Japan). cDNA was synthesized from 1 µg of DNase-treated total RNA from skin of wild-type (+/+), heterozygous (*L*/+) and homozygous (*L*/*L*) mice using SuperScriptTM III (Invitrogen Japan, Tokyo, Japan). Actb was used for normalization. Real-time quantitative PCR analysis confirmed the unchanged relative expression levels of the *Gsdma3* gene between wild-type and *Gsdma*$^{LacZ/LacZ}$ mice at P5.
