# Supplementary material for: Functional Conservation of Gsdma Cluster Genes Specifically Duplicated in the Mouse Genome
Source: G3 (Bethesda). 2013 Oct 1;3(10):1843–50. doi: 10.1534/g3.113.007393 (PMC3789809; doi:10.1534/g3.113.007393)
Supplement: Supporting Information [file supp_g3.113.007393_FigureS3.pdf]

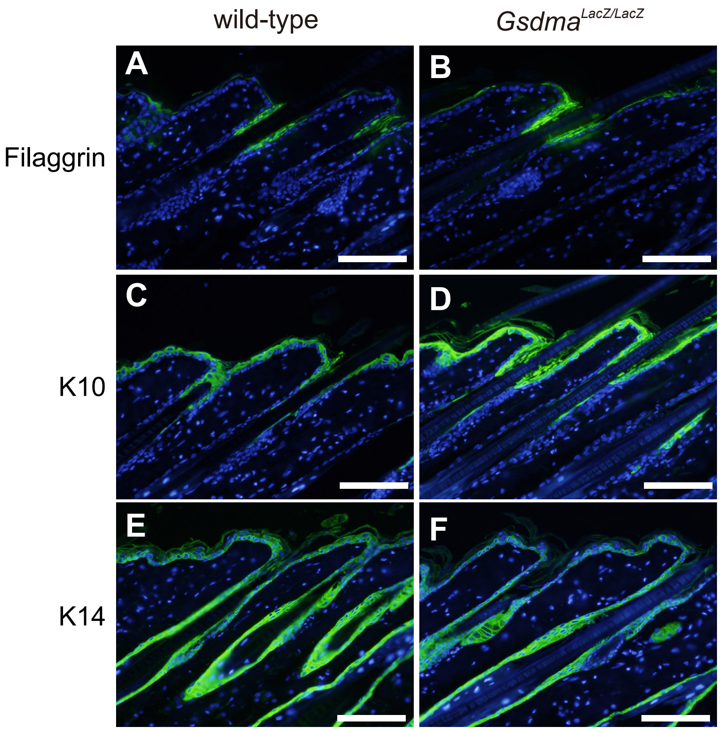

Figure S3 The expression of epidermal differentiation markers. Skin sections of wild-type (**A**, **C** and **E**) and *Gsdma*$^{LacZ/LacZ}$ mice (**B**, **D** and **F**) at 1 month of age. Filaggrin (1:100, Covance, Richmond, CA, USA) was used as a marker for the cornified cell layer (**A** and **B**). K10 (1:50, Covance) was used as a marker for the granular cell layer (**C** and **D**). K14 was used as a marker for the basal cell layer (**E** and **F**). Nuclear staining was performed by DAPI (Invitrogen Japan). Scale bars are 100 μm.
