# Supplementary material for: Functional Conservation of Gsdma Cluster Genes Specifically Duplicated in the Mouse Genome
Source: G3 (Bethesda). 2013 Oct 1;3(10):1843–50. doi: 10.1534/g3.113.007393 (PMC3789809; doi:10.1534/g3.113.007393)
Supplement: Supporting Information [file supp_g3.113.007393_FigureS4.pdf]

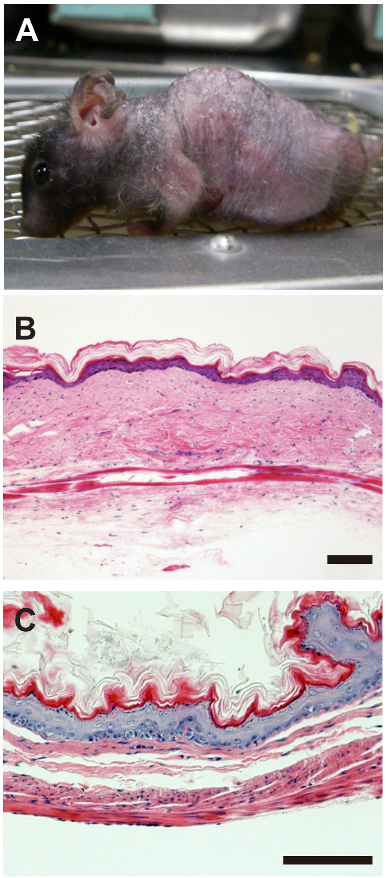

Figure S4. Phenotypes of a mouse with K5-*Gsdma* (A339T) transgene at 1 year of age. Macroscopic phenotype of a K5-*Gsdma* (A339T) transgenic mouse at 1 year of age (**A**). HE stained sections of skin (**B**) and cardia (**C**) of a K5-*Gsdma* (A339T) transgenic mouse at 1 year of age. Scale bars are 100 μm.
